# Force systems of direct printed and thermoformed aligners with different trimlines during rotational movements: an in vitro analysis

**DOI:** 10.1186/s40510-026-00634-1

**Published:** 2026-06-22

**Authors:** Emilia von Waldthausen, Lisa-Marie Mai, Philipp Meyer-Marcotty, Anja Quast, Jonas Q Schmid, Phillipp Brockmeyer, Wolfram Hahn, Bernhard Wiechens

**Affiliations:** 1 Department of Orthodontics, Universitätsmedizin Göttingen, Göttingen, Germany; 2 Department of Orthodontics, University of Münster, Münster, Germany; 3 Department of Oral and Maxillofacial Surgery, Universitätsmedizin Göttingen, Göttingen, Germany

**Keywords:** Direct printed aligners, Thermoformed aligners, Force decrease, Rotation, Gingival margin design, Orthodontics

## Abstract

**Background:**

The present study was conducted to quantify and compare the geometric accuracy and force application of thermoformed aligners (TFA) and direct-printed aligners (DPA) during mesial and distal rotation of a central upper incisor with a defined activation of 2°.

**Methodology:**

A 3D-printed model with a mounted central incisor was used to simulate 2° of rotational movement. Forces were recorded in three axes (Fx, Fy, Fz) over 60 minutes using a multi-axis force sensor. Three aligner types (thermoformed with straight trimline: TFAS, direct printed with straight trimline: DPAS, direct printed aligner with garlanded trimline: DPAG) were tested (n = 10 each). Vertical, transverse, and sagittal force components and their decrease were analyzed, and thickness measurements were taken.

**Results:**

Significant differences in resulting forces were observed between TFA, DPAS, and DPAG during both mesial and distal rotation. TFA generated significantly higher sagittal forces during mesial rotation (1.40 (0.30) N) compared to DPAS (-0.04 (0.05) N) and DPAG (0.11 (0.03) N; p < 0.001). Similar findings were observed for distal rotation (TFA 0.86 (0.16) N; DPAS -0.07 (0.06) N; DPAG 0.10 (0.09) N; p < 0.001). Transverse forces were generally lower, with only selected intergroup differences reaching significance. Time-resolved analysis revealed a pronounced initial force peak followed by continuous decay for TFA, while both DPA designs showed a gradual force increase with plateau formation.

TFA showed the most pronounced material loss, whereas directly printed aligners most closely matched the planned thickness. In mesial rotation, significant group differences were found for facial (TFA: 0.42 (0.01) mm; DPAS: 0.71 (0.15) mm; DPAG: 0.63 (0.06) mm), incisal (0.60 (0.02) mm vs. 0.76 (0.11) mm vs. 0.65 (0.06) mm) and palatal surfaces (0.63 (0.01) mm vs. 0.94 (0.17) mm vs. 0.80 (0.06) mm; all p < 0.001).

A comparable thickness pattern was observed for distal rotation.

**Conclusion:**

The results highlight the improved geometric accuracy and underscore the potential of 3D-printed aligners to improve force control and warrant further clinical investigation.

## Introduction

Over the past decades, the interest in clear aligner therapy has steadily increased, primarily driven by high patient acceptance due to superior esthetics and comfort compared with conventional fixed appliances [1]. The increasing demand has resulted in continuous innovation in digital workflows in dentistry. Computer-aided design and manufacturing (CAD/CAM) technologies have become an integral component of innovative orthodontic treatment approaches [2–5]. Within this digital transformation, additive manufacturing has emerged as a particularly promising development [4]. Conventional aligners require a multi-step production process, including thermoforming over a printed dental model prepared in advance. Recent advancements have enabled the direct 3D printing of aligners, eliminating several intermediate production steps [6]. This technological shift is considered a major milestone in aligner fabrication, offering not only economic and ecological advantages but also the potential for enhanced biomechanical precision. Directly 3D-printed aligners have been reported to exhibit a more homogeneous material thickness and improved fit in comparison with conventionally thermoformed aligners [7]. Furthermore, recent clinical data indicate that directly printed aligners are effective in managing mild to moderate malocclusions, achieving a significant reduction in malocclusion severity [8]. In contrast, the thermoforming process is unavoidably associated with thickness inconsistencies and material deformation, which may influence force delivery and compromise biomechanical predictability. The ability to digitally define aligner geometry and material distribution in directly printed aligners therefore represents a substantial advantage in terms of controlled force application [9]. From a clinical perspective, clear aligner therapy has demonstrated reliable outcomes particularly for anterior tooth movements. Rotational movements of maxillary central incisors, a frequent indication in aligner treatment, are generally considered predictable within certain limits, with rotations of up to approximately 40° reported to be clinically manageable [10].

Nevertheless, despite increasing clinical use and favorable treatment outcomes, the underlying biomechanical mechanisms remain insufficiently investigated. In particular, the magnitude and direction of forces generated during rotational movements have not been comprehensively quantified. Current literature highlights a relevant lack of data regarding the actual forces applied to teeth during clear aligner therapy, especially when comparing different manufacturing techniques [10, 11].

This knowledge gap is clinically relevant, as excessive or poorly controlled forces may adversely affect biological responses and treatment efficiency [12].

Therefore, the aim of the present in-vitro study was to investigate and compare the forces generated during rotational movements of the maxillary central incisors using conventionally thermoformed aligners and directly 3D-printed aligners. By providing experimental data on force characteristics associated with different manufacturing approaches, this study seeks to contribute to a better biomechanical understanding of aligner-mediated rotational tooth movement.

## Materials and methods

Force measurements were performed using a validated in-vitro test system [13, 14] consisting of a maxillary training model (Frasaco GmbH, Germany) with a modular maxillary central incisor (tooth 21) connected with a multi-axis force sensor (Nano 17, ATI Industrial Automation, USA). The model was digitized using an intraoral scanner (TRIOS 4, 3shape, USA).

Subsequently, software-assisted segmentation and preparation of the sample tooth, including drilling and model adaptation for sensor integration, were performed using 3D modeling software (Blender v4.0.2, Blender Foundation, Netherlands).

The gingival contour around tooth 21 was digitally reconstructed based on the surrounding teeth and designed to resemble a natural cervical anatomy. No intentionally increased undercuts were created. Small gaps needed for assembling the model were filled to prevent artificial retention effects.

The central incisor was programmed to undergo a rotational movement of 2° in either mesial or distal direction, representing a clinically relevant movement scenario.

The rotational movement was defined as a rotation about the long axis of tooth 21. The experimental setup was oriented such that the sensor’s z-axis was aligned with the long axis of the tooth. Consequently, the programmed rotation was performed approximately about the sensor’s z-axis, allowing a direct biomechanical interpretation of the recorded force components without the need for additional coordinate transformation.

**Fig. 1:**
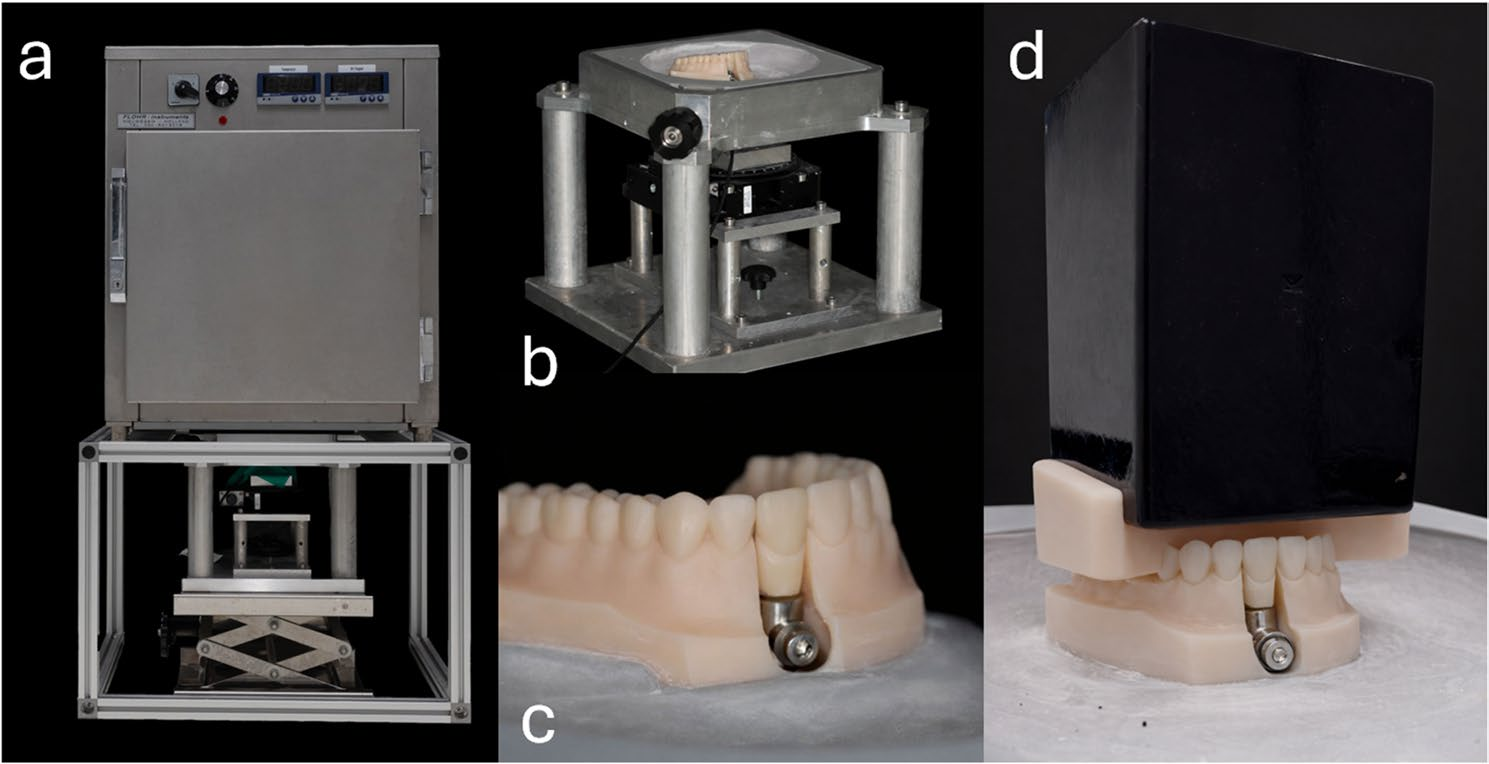
Test setup. Complete test setup with sensor unit with incubator (**a**), sensor unit (**b**), model with the separated tooth 21 (**c**) and the loading device (**d**)

After 3D printing of the investigation base, the measuring tooth, and the virtually designed mounting aid (OnyxCeph, Image Instruments, Germany), the setup was fixed in a resin base (Fig. 1b, c; GC Fujirock^®^ EP, GC Germany GmbH, Germany).

All measurements were conducted in an incubator (Fig. 1a; Flohr Instruments, Netherlands) at a constant temperature of 36.5 °C to simulate average intraoral conditions [15]. Artificial saliva was applied to further approximate the oral environment (Saseem, G. Pohl-Boskamp GmbH & Co. KG, Germany). Prior to each measurement, the inner surface of the aligners was coated with artificial saliva using a standardized spray application (two sprays per aligner), which was consistently performed for each test run.

**Fig. 2:**
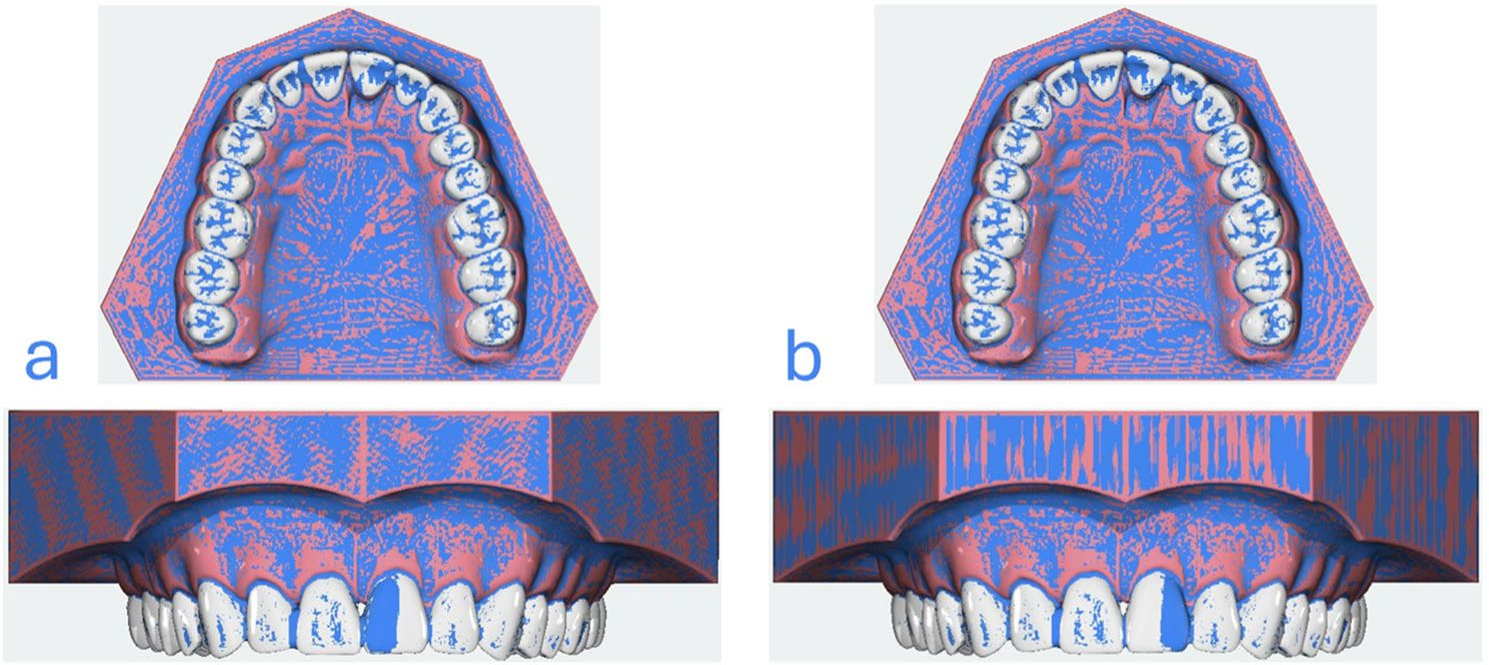
Superimposition of intended range of motion. Section (**a**) shows distal rotational movement, section (**b**) shows mesial rotational movement

The force sensor was calibrated according to the manufacturer’s specifications with an accuracy of 1% of full scale. After installation, another scan was performed for virtual reconstruction and planning of the tooth movement (Fig. 2; Aligner 3D, OnyxCeph, Image Instruments, Germany). In line with current clinical staging recommendations, a rotational movement of 2° mesially and distally was defined for tooth 21 [16].

For fabrication of the thermoformed aligners (TFA), an STL file of the modified dental arch was generated, specifying a vertical extension of 1–2 mm (Duran 0.75 mm, SCHEU-DENTAL GmbH, Germany). Planning of the directly printed aligners (DPA) was performed digitally using a straight (DPA_S_) as well as garland-shaped (DPA_G_) trimline design (Bitesplint 3D, OnyxCeph, Image Instruments, Germany). The garland-shaped trimline design corresponds to what is commonly described in the literature as a scalloped margin design. For clarity and internal differentiation between the investigated groups, the term “garlanded” is used throughout this study.

**Fig. 3:**
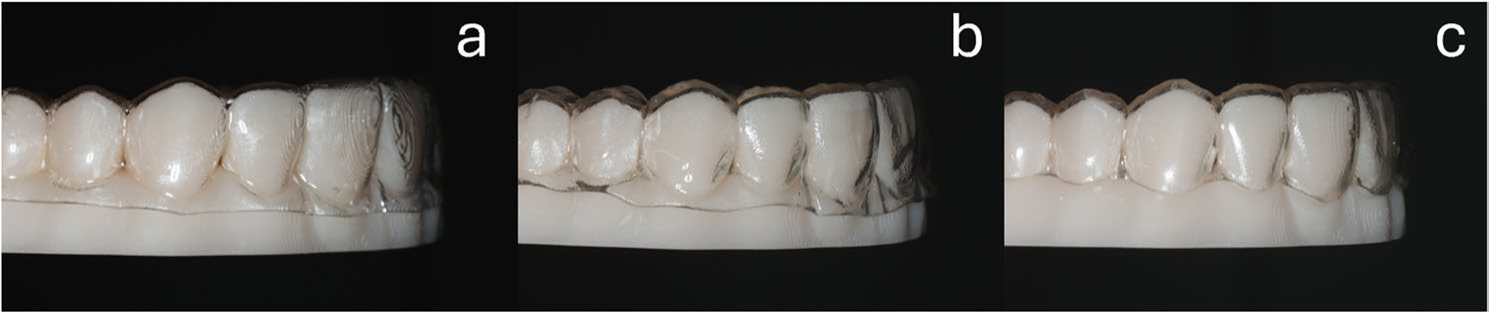
Tested samples. Illustration of aligners examined: TFA (**a**), DPA_S_ (**b**), and DPA_G_ (**c**)

The planned aligner thickness was 0.75 mm. The vertical extension of the straight-trimmed aligners was set to + 1.5 mm, whereas the garland-trimmed aligners were designed with a -1 mm extension. Corresponding STL files were generated and transferred to an external manufacturing facility (Fig. 3; Graphy Inc., Republic of Korea). As the aligners were produced using the in-house manufacturing process of the developer (TC-85DAC, Graphy Inc., Republic of Korea), regulatory-compliant production could be assumed.

Ten aligners were produced for each group (TFA, DPA_S_, DPA_G_). In view of the material properties of the directly printed aligners, force measurements were recorded at the following times (in minutes): 0, 1, 2, 3, 4, 5 and then at 5-minute intervals up to 60 min. Further measurements were conducted under a simulated swallowing load of 3 kg, based on previously reported physiological occlusal forces [17]. For this purpose, a hollow weight tray was designed (Blender v. 4.0.2, Blender Foundation, Netherlands) and a calibrated weight (Sport-Thieme GmbH, Germany) was placed exclusively on the designated support surfaces of the model (Fig. 1d). For comparison, an aligner from the TFA group was also evaluated according to the complete measurement protocol, while the remaining TFA samples were measured after 60 min.

**Fig. 4:**
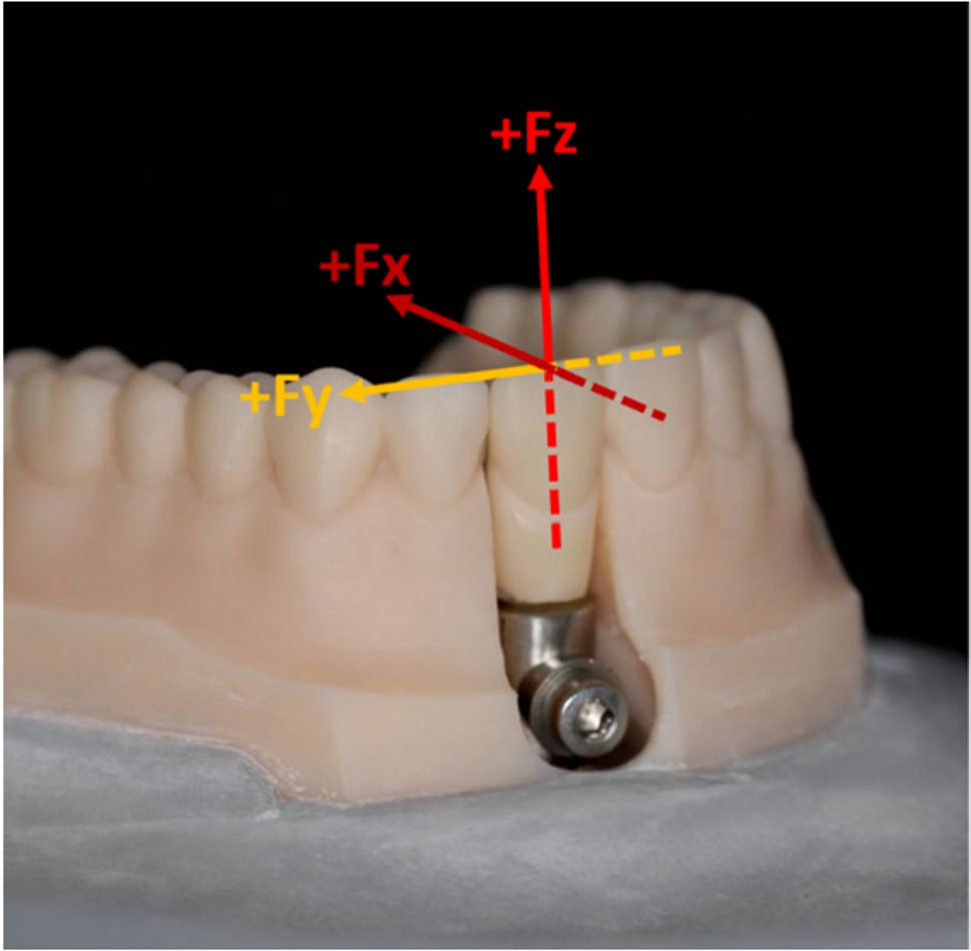
Force directions. Resulting force direction in the sagittal (Fx), transverse (Fy) and vertical (Fz) planes

The rigid sensor unit recorded the resulting force components in Newtons (N) along the sagittal (x), transverse (y), and vertical (z) axes. Positive values represented oral-directed forces in the sagittal plane, distal-directed forces in the transverse plane, and extrusive forces in the vertical plane while negative values indicated forces acting in the opposite directions (Fig. 4).

**Fig. 5:**
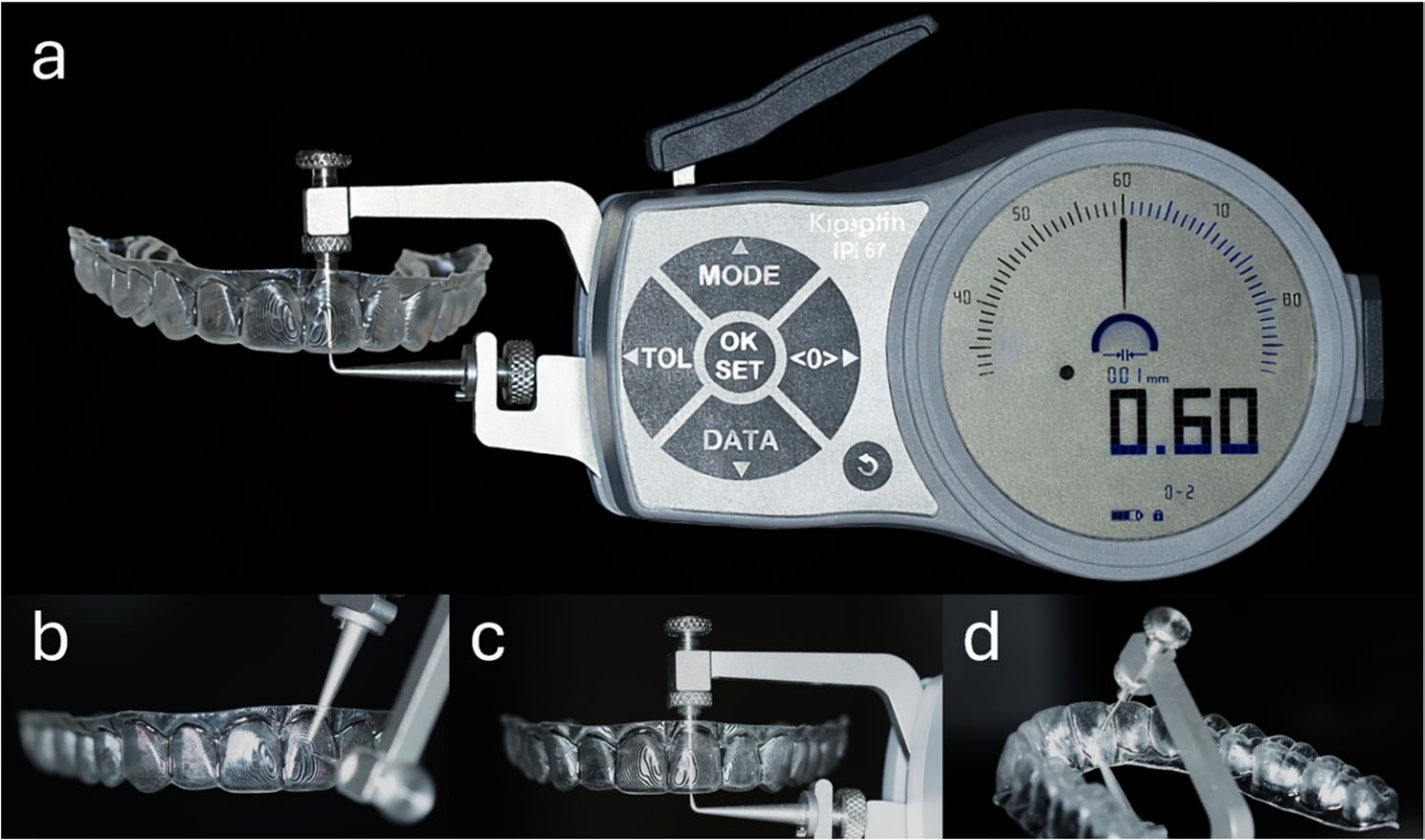
Tested samples and measuring device. Custom-made measuring device (**a**) and demonstration of measurement points: facial (**b**), incisal (**c**) and palatal (**d**)

Lastly, the thickness of all examined aligners was measured using an electronic external measuring device (K1 × 095, custom-made; Kroeplin Längenmesstechnik, Germany). Measurements were taken at the centre of the facial, incisal, and palatal surfaces of the respective tooth area (Fig. 5).

### Statistical analysis

First, data were tested for normality using the Shapiro-Wilk test. All results are presented as medians and interquartile ranges (IQR) and analyzed using the Kruskal-Wallis test with Dunn’s multiple comparisons. P-values were adjusted for repeated measurements using the Bonferroni method. Analyses were performed with GraphPad Prism 10 (v. 10.2.3) at α = 0.05.

## Results

**Fig. 6:**
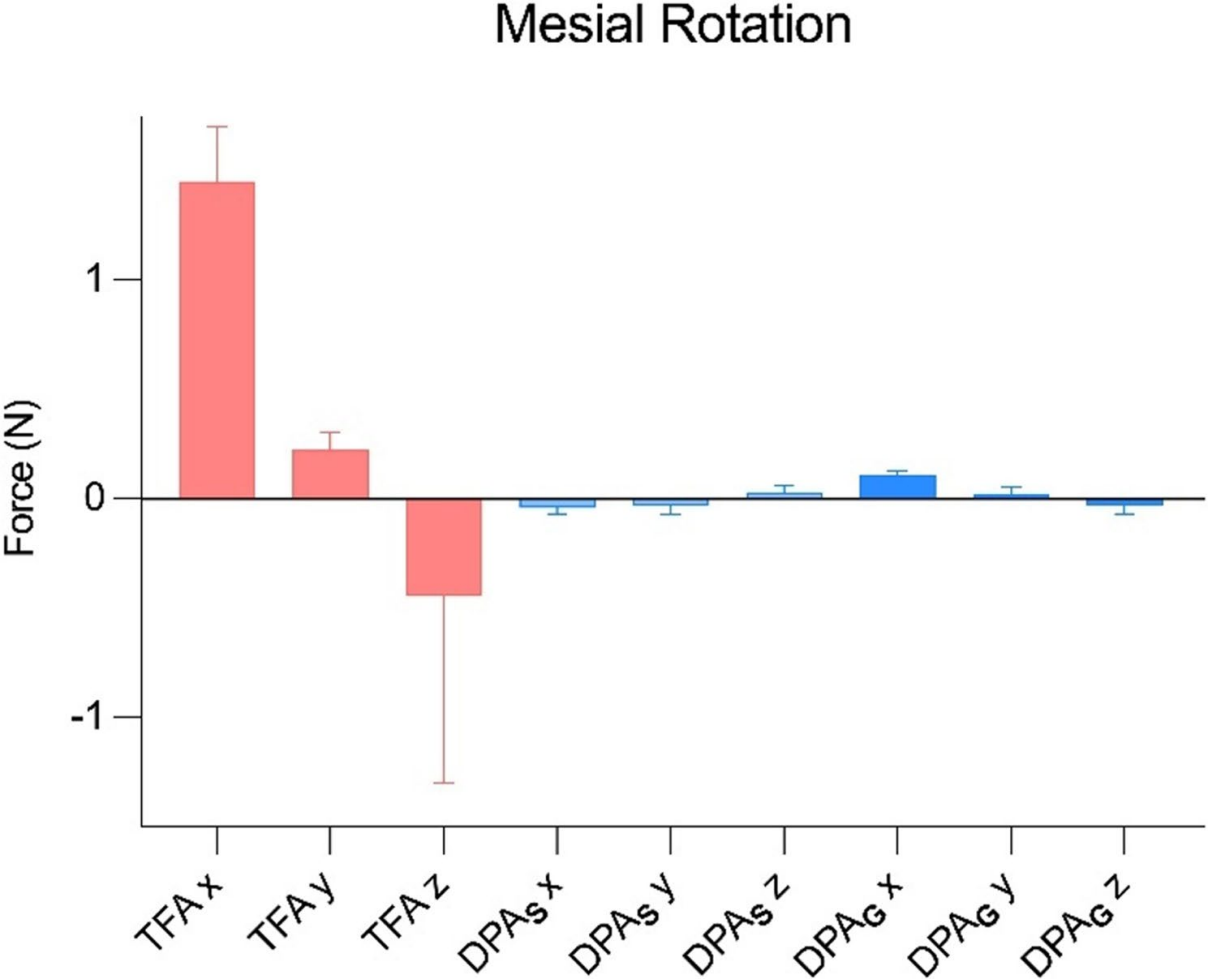
Resulting Forces during mesial rotation. Bar chart with error bars of resulting forces (Fx, Fy, Fz) during mesial rotation

**Fig. 7:**
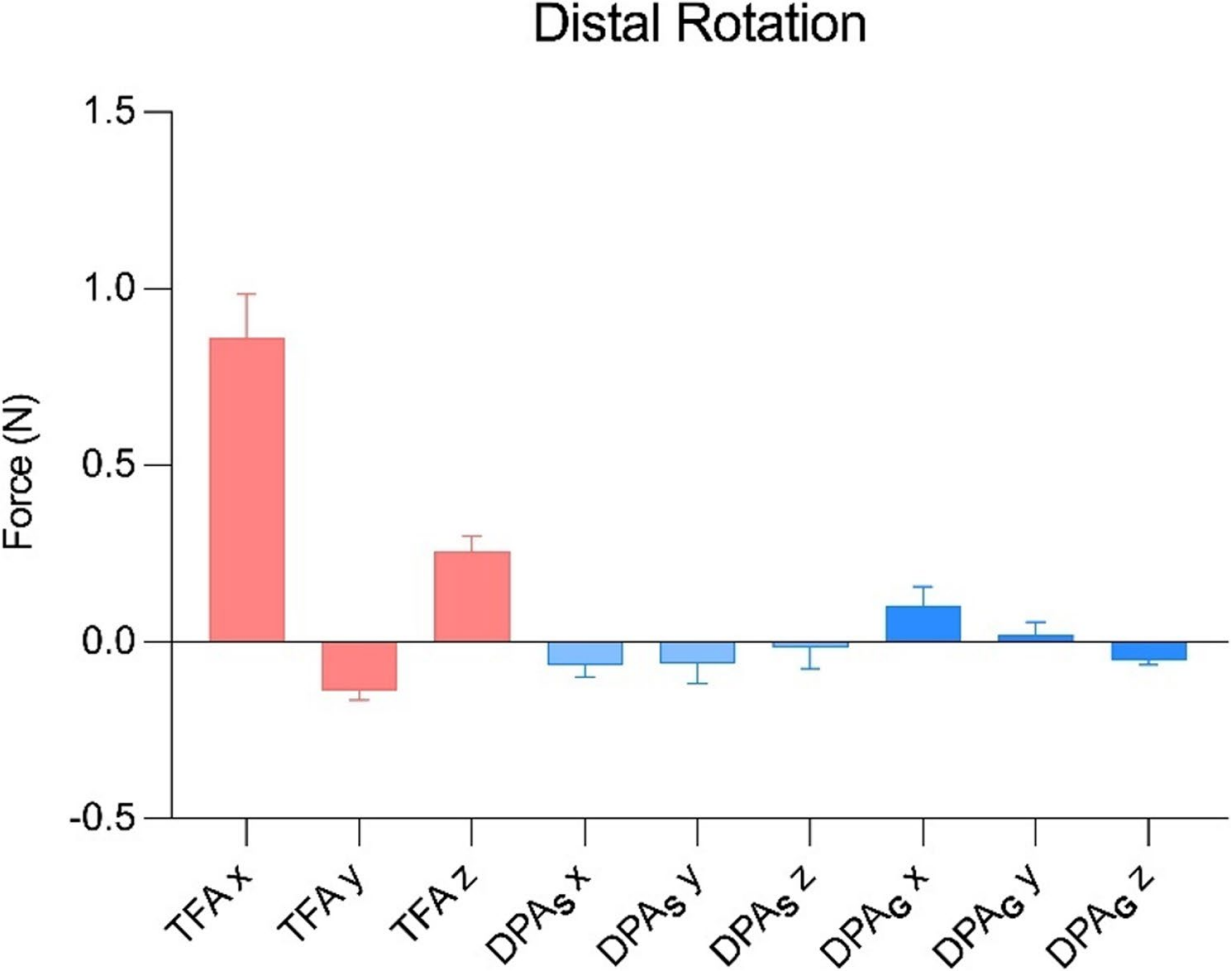
Resulting Forces during distal rotation. Bar chart with error bars of resulting forces (Fx, Fy, Fz) during distal rotation

For both mesial and distal rotation, significant differences in resulting forces were observed between the three groups (TFA, DPA_S_, DPA_G_) (Tables 1 and 2; Figs. 6 and 7).

**Table 1:** Descriptive and analytical statistics of resulting forces during mesial rotation

Resulting forces Mesial Rotation		TFA (*n* = 10)		DPA_S_ (*n* = 10)		DPA_G_ (*n* = 10)		Overall group comparison	Intergroup comparison		
				straight		garlanded		(Kruskal-Wallis)	TFA vs. DPA_S_	TFA vs. DPA_G_	DPA_S_ vs. DPA_G_
	Unit	Median	IQR	Median	IQR	Median	IQR	*p*	corr.*p*	corr.*p*	corr.*p*
sagittal (x)	N	1.40	0.30	-0.04	0.05	0.11	0.03	< 0.001	< 0.001	0.033	0.033
transverse (y)	N	0.22	0.33	-0.03	0.08	0.02	0.06	0.025	0.033	n.s.	n.s.
vertical (z)	N	-0.44	1.37	0.03	0.05	-0.03	0.04	0.028	n.s.	n.s.	0.027

Corresponding results of the Kruskal-Wallis test and Dunn’s multiple comparisons test between groups after 60 min are reported. Median values and interquartile ranges (IQR) in newtons (N) are presented; the significance level was set at *p* < 0.05. *ns*  Not significant

**Table 2:** Descriptive and analytical statistics of resulting forces during distal rotation

Resulting forces Distal Rotation		TFA (*n* = 10)		DPA_S_ (*n* = 10)		DPA_G_ (*n* = 10)		Overall group comparison	Intergroup comparison		
				straight		garlanded		(Kruskal-Wallis)	TFA vs. DPA_S_	TFA vs. DPA_G_	DPA_S_ vs. DPA_G_
	Unit	Median	IQR	Median	IQR	Median	IQR	*p*	corr.*p*	corr.*p*	corr.*p*
sagittal (x)	N	0.86	0.16	-0.07	0.06	0.10	0.09	< 0.001	< 0.001	0.033	0.033
transverse (y)	N	-0.14	0.21	-0.06	0.08	0.02	0.06	0.008	n.s.	0.013	0.038
vertical (z)	N	0.26	0.07	-0.02	0.08	-0.05	0.04	< 0.001	0.001	< 0.001	n.s.

Corresponding results of the Kruskal-Wallis test and Dunn’s multiple comparisons test between groups after 60 min are reported. Median values and interquartile ranges (IQR) in newtons (N) are presented; the significance level was set at *p* < 0.05. *ns*  Not significant

### Sagittal forces (Fx)

In the sagittal direction (x; vestibulo/oral), highly significant overall group differences were detected for mesial and distal rotation (both *p* < 0.001). During mesial rotation, TFA showed marked positive forces (median 1.40 N, IQR 0.30), whereas DPA_S_ showed slightly negative values (median − 0.04 N, IQR 0.05) and DPA_G_ small positive forces (median 0.11 N, IQR 0.03). Similar patterns were observed during distal rotation, with marked positive forces for TFA (median 0.86 N, IQR 0.16), small negative forces for DPA_S_ (median − 0.07 N, IQR 0.06) as well as small positive forces for DPA_G_ (median 0.10 N, IQR 0.09). Intergroup comparisons consistently revealed significant differences between TFA and DPA_S_, TFA and DPA_G_, and DPA_S_ and DPA_G_.

### Vertical forces (Fz)

In the vertical direction (z; intrusive/extrusive), significant overall group differences were observed for mesial (*p* = 0.028) and distal rotation (*p* < 0.001). During mesial rotation, TFA showed marked negative vertical forces (median − 0.44 N, IQR 1.37), whereas DPA_S_ and DPA_G_ showed values close to zero. During distal rotation, TFA showed positive vertical forces (median 0.26 N, IQR 0.07), while both DPA_S_ and DPA_G_ showed negative values again close to zero. Significant intergroup differences were primarily observed between DPA_S_ and DPA_G_ during mesial and TFA and the two DPA appliances during distal rotation.

### Transverse forces (Fy)

In the transverse direction (y; mesial/distal), significant overall group effects were found for mesial (*p* = 0.025) as well as distal rotation (*p* = 0.008). TFA tended to generate higher force values compared with DPA_S_ and DPA_G_. However, only selected intergroup differences remained significant, particularly between TFA and DPA_S_ during mesial and between TFA and DPA_G_ as well as DPA_S_ and DPA_G_ during distal rotation.

### Force decrease behavior

**Fig. 8:**
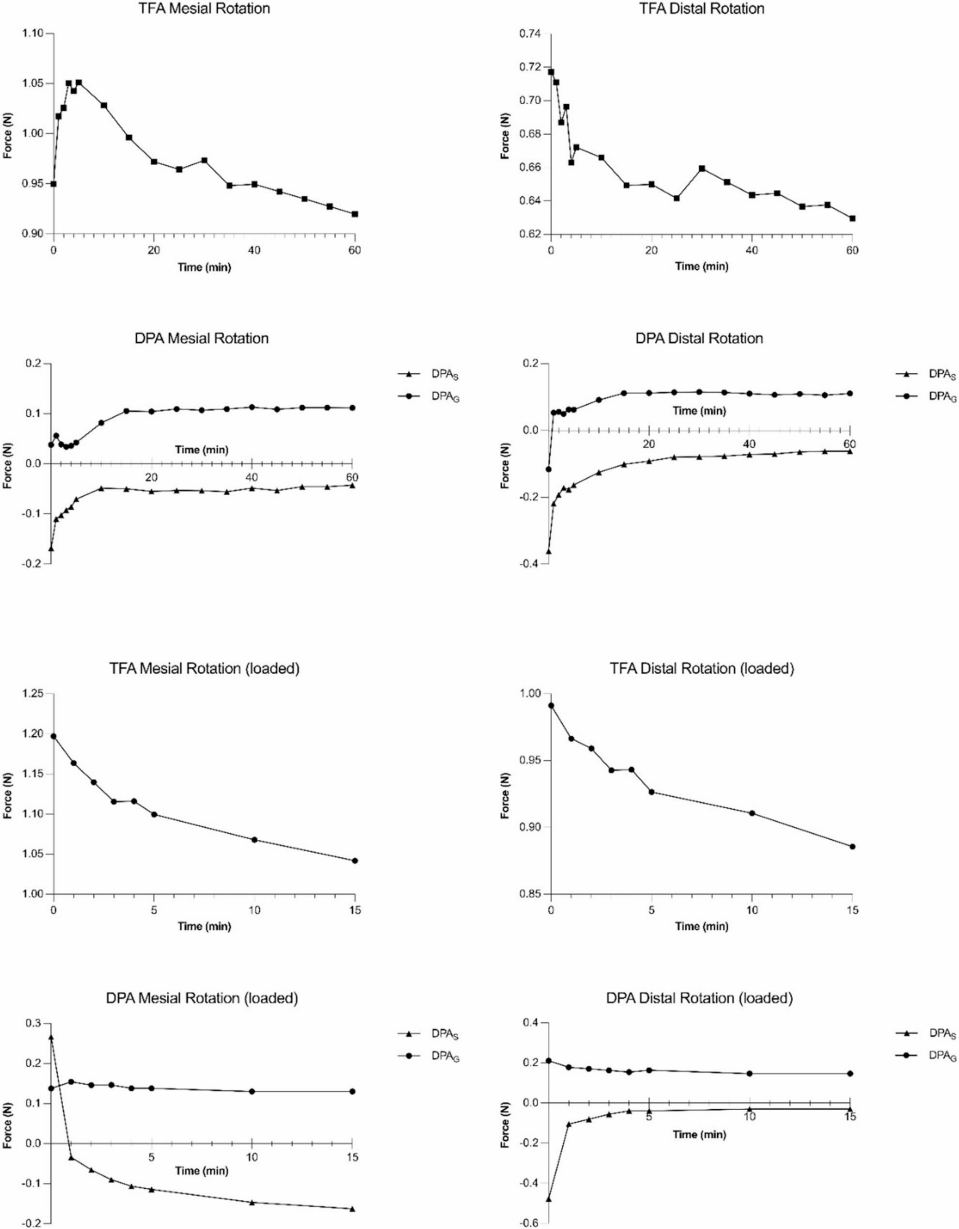
Time-dependent force development. Time-series line plots showing force development on the Fx-axis during mesial and distal rotation for thermoformed (TFA) and direct-printed aligners (DPA_S_ and DPA_G_), with and without occlusal loading

Time-resolved force analysis showed characteristic patterns for each aligner type. TFA exhibited a marked initial peak in force during both mesial and distal rotations, followed by a decline over the whole time. This pattern was consistent under unloaded and loaded conditions. In contrast, both DPA_S_ and DPA_G_ showed an initial increase in force followed by a plateau phase, with forces stabilizing in contrast to TFA. The plateau behavior was observed for both mesial and distal rotations and was consistent across both DPA designs (Fig. 8).

### Aligner thickness

Median aligner thicknesses at the facial, incisal, and palatal surfaces are presented in Tables 3 and 4. For both mesial and distal rotations, TFA showed the lowest thickness, while DPA_S_ exhibited the highest values across all measurement points.

**Table 3:** Aligner thickness during mesial rotation

Thickness Mesial Rotation		TFA (*n* = 10)			DPA_S_ (*n* = 10)		DPA_G_ (*n* = 10)		Overall group comparison	Intergroup comparison		
					straight		garlanded		(Kruskal Wallis)	TFA vs. DPA_S_	TFA vs. DPA_G_	DPA_S_ vs. DPA_G_
	Unit	Median	IQR		Median	IQR	Median	IQR	*p*	corr.*p*	corr.*p*	corr.*p*
Facial surface	mm	0.42	0.01		0.71	0.15	0.63	0.06	< 0.001	< 0.001	0.032	0.032
Incisal surface	mm	0.60	0.02		0.76	0.11	0.65	0.06	< 0.001	< 0.001	0.315 n.s.	0.017
Palatal surface	mm	0.63	0.01		0.94	0.17	0.80	0.06	< 0.001	< 0.001	0.020	0.080 n.s.

Corresponding results of the Kruskal-Wallis test and Dunn’s multiple comparisons test between groups. Median values and interquartile ranges (IQR) are presented. The significance level was set at *p* < 0.05. *n.s* Not significant

**Table 4:** Aligner thickness during distal rotation

Thickness Distal Rotation		TFA (*n* = 10)			DPA_S_ (*n* = 10)		DPA_G_ (*n* = 10)		Overall group comparison	Intergroup comparison		
					straight		garlanded		(Kruskal Wallis)	TFA vs. DPA_S_	TFA vs. DPA_G_	DPA_S_ vs. DPA_G_
	Unit	Median	IQR		Median	IQR	Median	IQR	*p*	corr.*p*	corr.*p*	corr.*p*
Facial surface	mm	0.43	0.02		0.73	0.07	0.66	0.02	< 0.001	< 0.001	0.028	0.043
Incisal surface	mm	0.61	0.01		0.74	0.06	0.66	0.04	< 0.001	< 0.001	0.064 n.s.	0.047
Palatal surface	mm	0.64	0.02		0.96	0.08	0.81	0.06	< 0.001	< 0.001	0.033	0.033

Corresponding results of the Kruskal-Wallis test and Dunn’s multiple comparisons test between groups. Median values and interquartile ranges (IQR) are presented. The significance level was set at *p* < 0.05. *n.s* Not significant

Kruskal-Wallis tests revealed highly significant overall group differences at all surfaces (*p* < 0.001). Dunn’s multiple comparisons indicated significant differences between TFA and both DPA_S_ and DPA_G_ at most surfaces. These patterns were consistent for both mesial and distal rotations. TFA have been observed to undergo a loss in thickness, a phenomenon that is particularly evident on the facial side. Conversely, DPA exhibited the most accurate alignment thickness in relation to the digitally designed aligner. While TFA exhibited the most significant loss of thickness on the facial surface, both DPA demonstrated the most substantial deviation in thickness on the palatal surface.

### Resulting forces under loaded conditions

**Fig. 9:**
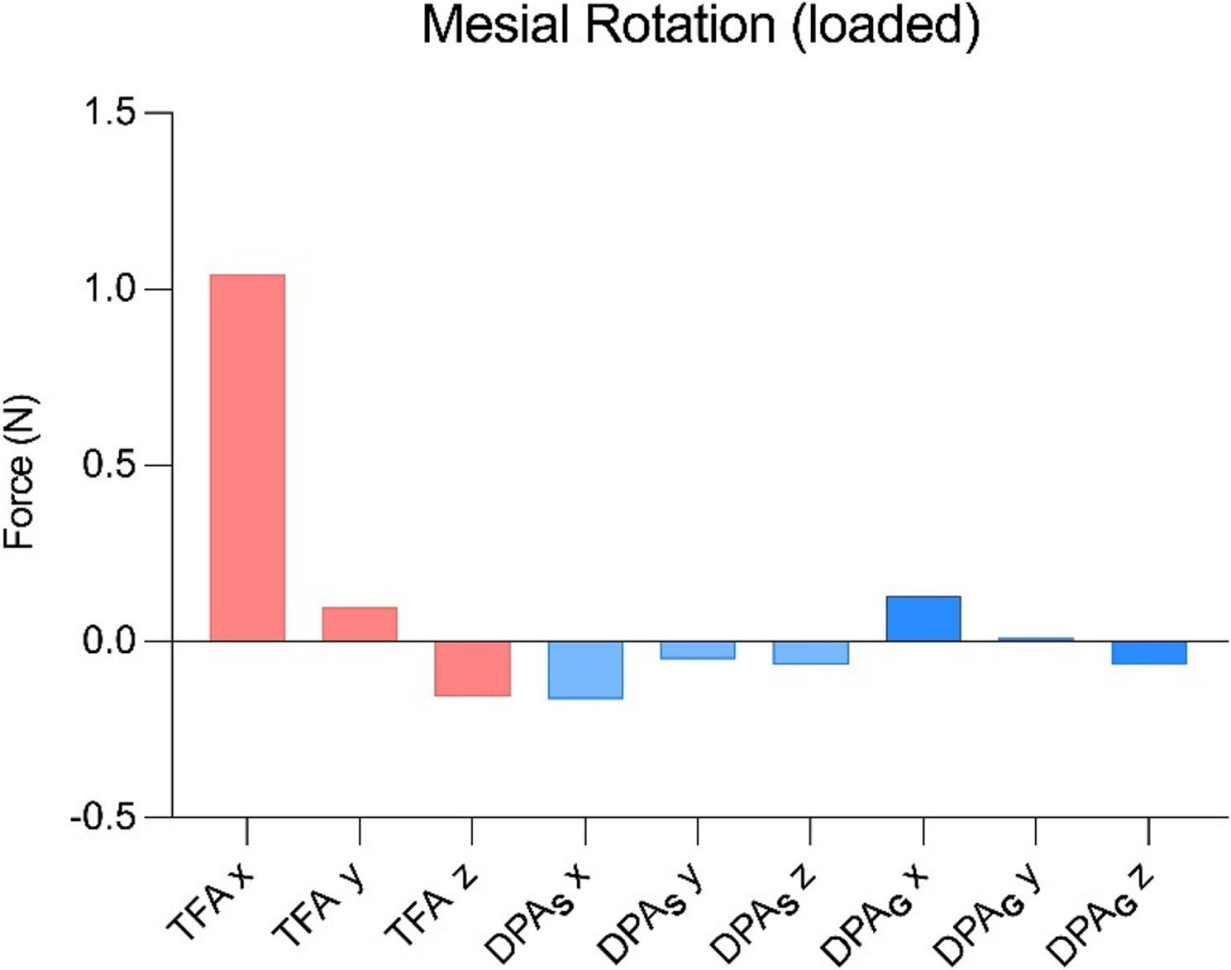
Resulting Forces during mesial rotation under loaded conditions. Bar chart with error bars of resulting forces (Fx, Fy, Fz) during mesial rotation

**Fig. 10:**
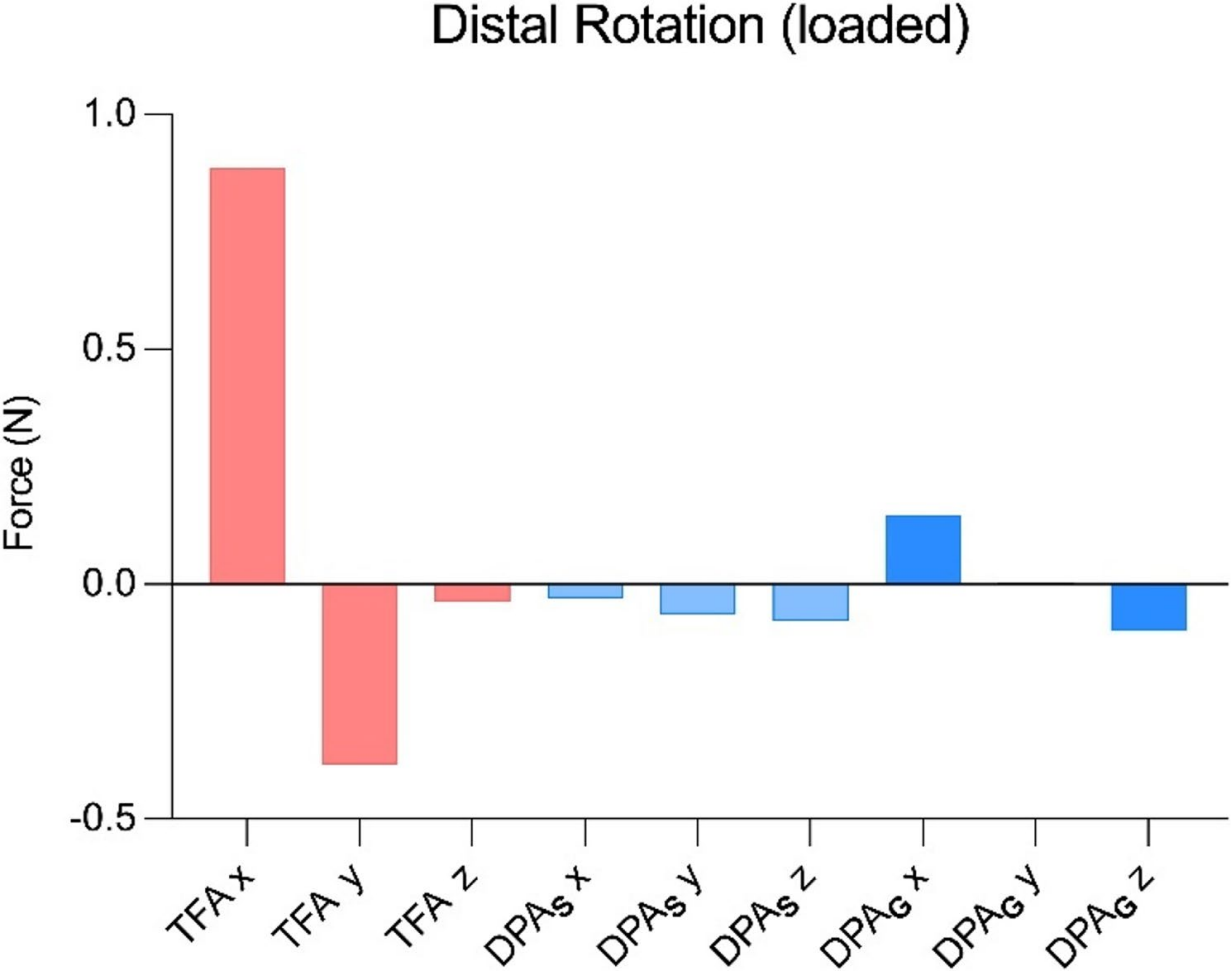
Resulting Forces during distal rotation under loaded conditions. Bar chart with error bars of resulting forces (Fx, Fy, Fz) during distal rotation

Under loaded conditions, one appliance per group was investigated. Therefore, the following results should be interpreted as a pilot observation and reported descriptively only (Tables 5 and 6; Figs. 9 and 10).

**Table 5:** Resulting forces during mesial rotation under loaded conditions

Resulting forces Mesial Rotation (loaded)	Unit	TFA (*n* = 1)	DPA_S_ (*n* = 1)	DPA_G_ (*n* = 1)
			straight	garlanded
sagittal (x)	N	1.04	-0.16	0.13
transverse (y)	N	0.10	-0.05	0.01
vertical (z)	N	-0.16	-0.06	-0.06

Descriptive analysis of resulting forces during mesial rotation under loaded conditions (3 kg for 15 min). Absolute force values in newtons (N) are presented; statistical analysis was not performed due to the limited sample size

**Table 6:** Resulting forces during distal rotation under loaded conditions

Resulting forces Distal Rotation (loaded)	Unit	TFA (*n* = 1)	DPA_S_ (*n* = 1)	DPA_G_ (*n* = 1)
			straight	garlanded
sagittal (x)	N	0.89	-0.03	0.15
transverse (y)	N	-0.38	-0.06	0.00
vertical (z)	N	-0.04	-0.08	-0.10

Descriptive analysis of resulting forces during distal rotation under loaded conditions (3 kg for 15 min). Absolute force values in newtons (N) are presented; statistical analysis was not performed due to the limited sample size

During mesial rotation, sagittal forces were positive for TFA (1.04 N) and DPA_G_ (0.13 N), whereas DPA_S_ showed negative values (-0.16 N). Transverse forces were low in all three groups, with values close to zero. Vertical forces were negative for all appliances, with the highest values observed for TFA (-0.16 N).

During distal rotation, sagittal forces remained positive for TFA (0.89 N) and DPA_G_ (0.15 N), with DPA_S_ showing values close to zero. In the transverse direction, TFA showed negative forces (-0.38 N), and DPA_S_ as well as DPA_G_ smaller values close to zero. Vertical forces were negative across all three groups, with DPA_G_ showing the highest values (-0.10 N).

## Discussion

The present study not only confirms but also extends previous findings demonstrating that directly printed aligners (DPA) generate more favorable force systems than conventional thermoformed aligners (TFA) during incisor derotation. TFA produced higher resulting forces, whereas both DPA designs exhibited markedly lower and more continuous force values.

Under unloaded conditions, TFA generated sagittal force values that were significantly higher than those observed for DPA_S_ and DPA_G_ during both mesial and distal rotation. These findings are consistent with earlier reports describing overactivation effects in thermoformed aligners [14, 18, 19]. Excessive forces are clinically undesirable, as they may increase the risk of going beyond the optimal biological force range for orthodontic tooth movement [12]. In contrast, the forces generated by DPA remained of small magnitude, suggesting a controlled as well as movement-specific force application.

Vertical force components further underline the biomechanical disadvantages of TFA. While DPA_S_ as well as DPA_G_ exhibited vertical forces close to zero during mesial and distal rotation, TFA generated marked intrusive or extrusive force values depending on the direction of rotation. Since vertical tooth movement is typically not intended during isolated derotation, such force values represent undesirable side effects, described in rotational movement using TFA before [14, 19]. The barely detectable vertical forces in the DPA groups supports the hypothesis that material and processing play a critical role in force vector control [20].

Referring to TFA, attachments are commonly employed to mitigate these unwanted force components [21]. The apparent absence of such side effects with directly printed aligners may therefore reduce the necessity for additional attachments in clinical practice.

Future studies should investigate the combined influence of composite attachments and aligner material properties on force delivery and rotational control. While attachments have been shown to improve force transmission in aligner therapy [22] directly printed aligners with shape memory properties may achieve more consistent fit and force delivery even without attachments due to their elastic recovery behavior and dimensional stability [23, 24]. These interactions warrant further evaluation under long-term and clinically representative conditions.

In the transverse direction, TFA still tended to produce higher force values compared to DPA. Although not all intergroup comparisons reached statistical significance, the overall pattern indicates a less selective force system for TFA, with DPA showing improved force control. Reduced transverse side effects are clinically relevant, as unwanted forces may contribute not only to root tipping or anchorage loss but also to root resorptions [12].

The descriptive results under loaded conditions support the unloaded findings, with TFA continuing to generate higher sagittal and transverse forces and DPA_S_ and DPA_G_ showing low force values in most directions. Although statistical analysis was not performed due to the limited sample size, these observations suggest that the unfavorable force characteristics of TFA are not only related to seating behavior but may be linked to the thermoforming process as well as material properties.

In this context, previous investigations have demonstrated that thermoformed aligners exhibit significantly greater thickness variations compared to directly printed aligners, with pronounced local thinning depending on aligner region and forming conditions [9]. Such inconsistencies in thickness are likely to contribute to variability in stiffness and force transmission. In contrast, directly printed aligners show a higher predictability of the planned thickness distribution, which may promote more uniform biomechanical behavior [25]. The thickness measurement results of the present study support this hypothesis. The TFA were observed to undergo a loss in thickness, the extent of which varied depending on the measured area. This change in material thickness can be attributed to the conventional thermoforming process, precluding the possibility of maintaining a constant material thickness across the entire aligner [26]. In contrast, both DPA designs exhibited values that remained closer to the digitally designed aligner thickness. An advantage, which is supported by previous studies, which have shown that DPA exhibit improved geometric trueness and enhanced fit accuracy [9, 25, 27]. A notable advantage of digital design is its ability to specify different thicknesses within an aligner, for instance, to provide specific support for the physical movement of a tooth [28].

Furthermore, DPA have been shown to exhibit superior fit accuracy compared to TFA, resulting in a more reproducible aligner-tooth interface [28, 29]. Improved fit may reduce uncontrolled preload and seating-related force peaks, thereby contributing to the consistently lower force magnitudes observed for DPA_S_ and DPA_G_ in the present study. From a material-mechanical perspective, these findings are further supported by evidence that thermoformed aligners tend to deform plastically and irreversibly under load, whereas directly printed aligners predominantly exhibit reversible elastic behavior [27]. Such irreversible deformation in TFA may amplify force variability during loading and unloading cycles. Such deformations hypothetically contribute to a loss of the initiated forces through the irreversible loss of their shape when the aligners are repeatedly removed and reinserted [30]. In contrast, the elastic behavior of DPA may be hypothetically linked to the pronounced shape-recovery capability of the TC-85 material, for which a substantial shape-memory effect has been demonstrated, with near-complete form recovery after defined time intervals [24].

In conclusion, these material- and process-related characteristics provide a mechanistic explanation for the more stable and predictable force behavior observed in DPA.

Time-resolved analysis of the force patterns revealed that thermoformed aligners exhibited a pronounced initial force peak during both mesial and distal rotations, followed by a marked decline over the observation period of one hour. This behavior was consistently observed under both unloaded and loaded conditions and indicates a rapid force decay after initial insertion. Such force patterns are well documented for thermoformed aligners and have been attributed to stress relaxation, material creep, and irreversible deformation processes occurring shortly after seating. Previous investigations have demonstrated that TFA tend to generate high initial forces immediately after insertion, followed by a substantial force reduction within a short time frame, particularly during rotational and tipping movements [30, 31].

In contrast, both directly printed aligner designs showed a distinctly different temporal force behavior. DPA_S_ and DPA_G_ exhibited an initial increase in force after insertion, followed by a plateau phase in which force levels remained relatively stable over the entire one-hour observation period. This plateau behavior was consistently observed for both mesial and distal rotations and across both DPA designs, indicating a more mechanically stable and predictable force application during rotational movements. This stable force behavior may be associated with the shape memory properties of directly printed aligner materials. For the TC-85 resin used in the present study, a pronounced shape-recovery behavior has been demonstrated, with increasing restoration of shape over specified time intervals [24].

This observed difference between TFA and DPA force patterns can be explained by their distinct manufacturing processes and material behaviors. During vacuum thermoforming, TFA are subjected to heat-induced expansion, causing uneven reductions in thickness of up to 50%, particularly on convex surfaces such as the buccal regions [9]. This process also leads to local material degradation, including altered water absorption, reduced surface hardness, and increased stress relaxation [32].

In contrast, directly printed aligners are manufactured additively with digitally controlled wall thickness, resulting in greater overall dimensional accuracy. A key material difference is the shape-memory property of the TC-85 resin used for DPAs. Lee et al. (2022) [24] demonstrated that this resin achieves 96% shape recovery at 37 °C within 60 min, whereas conventional PETG shows no such recovery. This allows DPAs to gradually restore their programmed geometry and maintain more consistent force over time. TFAs, relying on irreversible elastic deformation, show pronounced stress relaxation, with force drops of 54–62% within 24 h [33]. Consequently, despite higher thickness variability, DPAs deliver more consistent force profiles, in line with our 60-minute measurement data.

Regarding the aligner margin design, both DPA groups performed biomechanically superior to TFA. Differences between DPAS and DPAG indicate that also the trimline geometry affects force values. DPA_S_ exhibited more neutral force values, whereas the DPA_G_ showed slightly higher force components in selected directions in general. Previous studies also demonstrated that different margins influence aligner grip, force transmission as well as deformation behavior [24, 34].

However, both DPA designs maintained substantially lower force values than TFA, underlining that the manufacturing approach itself plays also a dominant role.

Within the DPA groups, an additional observation is of particular interest. In certain instances, force components were detected that acted in the opposite direction to the planned tooth movement. According to mesial and distal rotation, both the TFA and DPA_G_ models exhibit positive force values oriented in the palatal direction along the Fx axis. In contrast, the DPA_S_ model displays negative forces directed towards the vestibular direction. Comparable observations have already been reported in previous investigations analyzing tipping movements with directly printed aligners [35]. The present study extends these observations to rotational movements, indicating that this phenomenon is not limited to tipping but may represent a more general biomechanical characteristic of DPA systems.

Previous investigations have shown that TFA fabricated from single-layer PETG materials are likely to generating excessive initial forces [14, 18], while multilayer thermoforming foils have been introduced to improve force stability [20], yet are still limited by the limitations of the thermoforming process. Material thinning limited geometric control as well as residual stresses limit precise control over force systems. In contrast, DPA allow for direct control over not only aligner geometry and thickness distribution but also margin design, enabling more predictable and movement-specific biomechanics [36].

From a clinical perspective, the significantly lower force values observed for DPA suggest a lower risk of unintended tooth movements as well as biological overload. Excessive force application, as observed for TFA, has been associated with delayed tooth movement and a higher risk of root resorption [12]. These findings suggest that the force characteristics of DPA could enhance treatment outcome predictability.

However, the absolute force magnitudes measured in the present study must be interpreted with caution when transferring the findings to clinical conditions. Due to the rigid experimental setup without simulation of the periodontal ligament, the recorded forces are likely to overestimate the actual forces acting in vivo. Therefore, while the comparative differences between aligner types and the observed force patterns over time remain valid, the absolute force values cannot be directly translated into clinically relevant thresholds.

### Limitations

Several limitations must be acknowledged. Under loaded conditions, only one aligner per group was evaluated, limiting the ability to generate statistically robust conclusions. Furthermore, the investigation of torques in relation to rotational movement, was not examined. The present study examined two distinct rotational movements within a test setup, utilizing various aligner thicknesses. In order to perform a physically accurate calculation of the torque, it is essential to define the point of force application. It has been demonstrated that this point shifts diagonally during rotational movements, resulting in a greater shift of the center of rotation with increased aligner thickness [37].

Although moment vectors were recorded at the sensor origin, a clinically meaningful interpretation was not feasible due to the absence of a defined center of resistance in the in vitro setup. Further research could be directed towards an investigation within the framework of the same aligner thicknesses with the same rotational movement to compare the torques that occur.

In addition, the force systems were evaluated over a short observation period of one hour only. Therefore, the results should be interpreted as an early observation, as clinically relevant force decay and material-related changes are known to occur over longer time periods. Future studies should investigate aligner behavior under load over extended observation times to better reflect clinical conditions.

Furthermore, the in vitro design does not account for intraoral factors like temperature fluctuations and the biological variability of the periodontal ligament. Although artificial saliva was applied, it cannot fully replicate the complex properties and continuous flow conditions of natural saliva [38]. Nevertheless, the standardized experimental setup allows for a direct biomechanical comparison providing valuable insights into the influence of aligner manufacturing and design on force systems.

A further limitation is that the gingival and cervical region of the model was simplified in its digital reconstruction. Although care was taken to approximate a natural cervical anatomy and avoid intentionally increased undercuts, minor deviations from true intraoral conditions cannot be excluded. In the presence of locally increased retention, this could theoretically result in elevated force transmission, as previously shown for enhanced retentive elements designed to increase aligner-tooth coupling [39].

## Conclusion

In summary, this study demonstrates that TFA generate significantly higher and less controlled force systems during incisor derotation compared to DPA. Both DPA designs showed biomechanically more favorable force characteristics. These findings further highlight the potential of 3D printing technology to improve force control in aligner therapy and suggest the need for further clinical studies, particularly regarding the behavior of aligners under load during rotational movements.

## Data Availability

All data generated or analysed during this study are included in this published article.

## Abbreviations

DPA: Direct-Printed Aligner
DPA_S_: Direct-Printed Aligner with Straight Trimline
DPA_G_: Direct-Printed Aligner with Garlanded Trimline
Fx: Sagittal Force Component
Fy: Transverse Force Component
Fz: Vertical Force Component
TFA: Thermoformed Aligner
